# How effectively has a Just Culture been adopted? A qualitative study to analyse the attitudes and behaviours of clinicians and managers to clinical incident management within an NHS Hospital Trust and identify enablers and barriers to achieving a Just Culture

**DOI:** 10.1136/bmjoq-2022-002049

**Published:** 2023-01-26

**Authors:** Adam Tasker, Julia Jones, Simon Brake

**Affiliations:** 1 Warwick Medical School, University of Warwick, Coventry, UK; 2 Rosalind Franklin Laboratory, UK Health Security Agency, London, United Kingdon; 3 Research & Develpment Division, South Warwickshire Universty Foundation NHS Trust, Warwick, UK

**Keywords:** safety culture, patient safety, human factors, health policy, human error

## Abstract

**Objectives:**

Just Culture aims to improve patient safety by examining the organisational and individual factors that contribute to adverse events, enabling corrective action so that errors are not repeated. This qualitative study aims to: (1) analyse whether the attitudes and behaviours of clinicians and managers are aligned with a Just Culture; (2) identify barriers and enablers to an organisation adopting a Just Culture.

**Methodology:**

This qualitative study used interviews and observation of Trust meetings to elicit the attitudes and behaviours of staff. Semistructured interviews were conducted with 13 doctors of all grades, 5 medical students and 2 managers. Five meetings that reviewed clinical incidents and mortality were observed. This was done in a single Hospital Trust in the Midlands, England. Data were thematically analysed using directed and inductive approaches.

**Results:**

There is evidence of a fair incident management process within the Trust; however, there was no agreed vision of a Just Culture and the majority of the staff were unfamiliar with the term. Negative perspectives relating to clinical incidents and their management persist among staff with many having insecurities regarding being the subject of an investigation and doubts about whether they drive improvement.

**Conclusion:**

This paper examines the significance of these findings and provides recommendations which may have application within other healthcare organisations. Major recommendations include (1) Just Culture: define an agreed vision of what Just Culture means to the Trust; (2) investigations: introduce incident management familiarisation training; (3) Learning Culture: increase face-to-face communication of outcomes of investigations and incident review; (4) investigators: establish an incident investigation team to improve the timeliness and consistency of investigations and the communication and implementation of outcomes.

**WHAT IS ALREADY KNOWN ON THIS TOPIC**

- A Just Culture aims to improve clinical incident review and investigation. Though many healthcare systems have looked to implement a Just Culture, there is little research into barriers and enablers of adoption.

**WHAT THIS STUDY ADDS**

- This study analyses clinicians’ attitudes and behaviours regarding a Just Culture and identifies some of the enablers and barriers to its adoption.

**HOW THIS STUDY MIGHT AFFECT RESEARCH, PRACTICE OR POLICY**

- In this paper, we make four recommendations to improve clinical incident review in a healthcare setting.

## Background

Commitment to quality of care is a core value of the National Health Service (NHS); however, over the past two decades cultures found within NHS trusts have been cited as contributing to major failings of patient safety and care.^1–3^ One aspect of problematic cultures observed is the presence of a Blame Culture within which blame, fear and secrecy are dominant when clinical incidents are investigated. The 2000 Department of Health report ‘An Organisation with Memory’^4^ attempted to address problematic cultures by adopting a Safety Culture based on work by Reason.^5^ The four components of Reason’s Safety Culture are a Just Culture, Reporting Culture, Learning Culture and a Flexible Culture. While interventions have been placed to improve the Reporting and Learning Cultures, such as Freedom to Speak Up Guardians^6^ and DATIX,^7^ the perception of a Blame Culture remains.^8 9^ Initiatives to counter this over time have included attempts to establish a ‘no-blame culture’, followed by a ‘fair blame culture’ and an ‘open and fair culture’.^10^ Despite these initiatives, both the Williams Review^8^ and the Hamilton Review^9^ found that the perception of a culture that seeks to blame rather than understand and learn persists, creating a sense of fear and cautiousness among clinicians. The most recent NHS Staff Survey^11^ also demonstrated reluctance of staff to engage with safety systems with 25.1% of respondents insecure about raising a concern regarding unsafe clinical practice and 40.6% lacking confidence that their organisation would address their concern. Worse patient care results as opportunities are missed to amend procedures and prevent similar incidents. The presence of a culture of bullying and intimidation was recently highlighted as contributing to the performance of Nottingham University Hospitals NHS Trust’s maternity services.^12^

The NHS renewed its focus on establishing a Just Culture through the Just Culture Guide^13^ and Being Fair Report.^14^ These publications reference two theories: Reason’s Safety Culture^5^ which highlights the importance of analysing and addressing both organisational and individual factors that contribute to an incident; and Dekker’s Restorative Just Culture^15^ which focuses on repairing relationships and meeting the needs of affected parties. The NHS’ use of these two distinct theories in its main guides has the potential to generate confusion regarding what is meant by the term Just Culture. This may, however, be beneficial if it prompts debate, enabling staff within an organisation to determine what Just Culture means to them and take ownership of the meaning they ascribe.^16^ The Being Fair Report^14^ also highlighted the importance of the study of human factors to improve the management of clinical incidents. Human factors in healthcare has been defined as ‘Enhancing clinical performance through an understanding of the effects of teamwork, tasks, equipment, workspace, culture and organisation on human behaviour and abilities and application of that knowledge in clinical settings’.^17^

In this article, we report the results of a qualitative study, which used interviews and ethnography to examine the attitudes and behaviours of staff and students at an NHS Hospital Trust. The work aimed to:

- Analyse the extent to which participants’ attitudes and behaviours to clinical incidents were aligned with those promoted by a Just Culture.
- Identify the barriers and enablers to an organisation adopting a Just Culture.

This study was conducted during the COVID-19 pandemic, during a period of additional pressure that included staff shortages and late presenting patients.

## Methodology

This study was conducted at a single NHS Hospital Trust in the Midlands. Site selection was based on the presence of a streamlined ethics approval process that existed between Warwick Medical School (WMS) and the Trust, which enabled research approval to be gained within set timelines. Researchers were given permission to advertise the research project within the Trust, conduct interviews with staff and observe meetings held via MS Teams that reviewed clinical incidents and mortality. Researchers were not given access to Trust policies regarding clinical incident management but they did meet with the Trust’s Head of Clinical Effectiveness who described incident management process. This informed researchers’ selection of meetings to observe and identified managers with key responsibilities for patient safety.

Twenty semistructured interviews were conducted with clinicians and managers (see online supplemental file 1 for the interview schedule). The research project was advertised at the Trust via trust-wide emails and flyers were handed out at handover meetings, and at WMS via forum posts. Stratified voluntary response sampling was used to recruit clinicians by seniority (table 1). Clinicians included all doctors working at the Trust and all third and fourth year medical students (MS) at WMS. Purposeful sampling was used to recruit two managers who were involved in enabling the patient safety culture in the Trust. The two managers were approached via email. Our exclusion criterion for all interviewees was ‘anyone with less than six weeks’ experience at the Trust’. Our target sample size was 15 doctors, 5 MS and 2 managers. This sample was the smallest that would capture a range of perspectives and reduce the risk of failing to identify outliers. Unfortunately, three participants dropped out as we were unable to arrange a suitable time for interview.

10.1136/bmjoq-2022-002049.supp1 Supplementary data

**Table 1:** Number of interviews conducted by seniority

Grade	Recruited	Target
Manager (MAN)	2	2
Consultant (CONS)	4	5
ST3/IMT3+ (registrar—REG)	5	5
FY1-ST2/IMT2 (recently qualified—RQ)	4	5
Medical student (MS)	5	5

The decision to focus primarily on medical doctors was made for two reasons. First, this cohort’s views were strongly expressed by the Williams and Hamilton reviews. This presented the opportunity to analyse whether interviewees still hold concerns similar to those reported by these reviews. Second, the sample size prevented effective stratified sampling by seniority and profession had participants from multiple healthcare professions been interviewed.

One-to-one interviews lasting 21–75 min were conducted and recorded via MS Teams. The interview schedule examined knowledge of a Just Culture in the closing questions to avoid biasing interviewees’ responses to questions relating to workplace culture and incident management. It was pilot tested once prior to interviews.

The researcher was in a quiet room alone; however, there were no stipulations for the location of the interviewee and some chose to hold their interviews in an office with colleagues present. The interviews were auto-transcribed by MS Teams, and transcripts were corrected by the member of the research team who did not conduct the interview. The transcript was not returned to the participant for comment or correction. The recording was then deleted. Repeat interviews were not carried out.

Three directorate-level Morbidity and Mortality meetings (M&Ms) and two trust-level incident review (TLIR) meetings were observed to analyse the attitudes and behaviours of attendees. The Trust allocates reported incidents to the most appropriate directorate and an investigator is selected from any member of staff within that directorate. Incidents with a moderate or severe risk of harm or death and recurrent incident themes are discussed at TLIRs. Any incident that is deemed to meet the Serious Incident Framework criteria at the TLIR receives executive oversight. TLIRs also discuss the results of root cause analyses (RCA) of incidents during which the lead investigator (RCA Lead) presents their findings.

Given the narrow data collection window, convenience sampling was used when selecting meetings for observation. When multiple M&Ms were available, a stratified approach was taken to observe meetings from a variety of directorates. M&Ms conducted by the Acute Medicine, Women’s and Child Health and Intensive Therapy Unit directorates were observed. Our exclusion criteria were:

- Meetings that did not contain an element of incident or mortality review.
- Meetings where any attendee was unwilling to participate in our observation.

Two researchers independently observed meetings via MS Teams and took ethnographic field notes of how staff analysed and presented case studies during the meetings. These notes were then reflected on immediately after the meeting.

A review of relevant NHS guidance and academic literature^1 4 5 8 9 13–15 18 19^ was used to construct the interview schedule and directed codes. Questions explored interviewees’ perceptions of the Trust’s culture and how it related to a Just Culture using the traits summarised in table 2. Data were thematically analysed using directed analysis to assess whether attitudes and behaviours were aligned with a Just Culture. Inductive analysis of interview transcripts was used to understand the enablers and barriers to improving a Safety Culture. No themes were identified in advance of the interviews. Two researchers independently coded interview transcripts and field notes using NVivo. Inductive codes were compared twice during data collection and once after data collection. All interview transcripts were then recoded. These codes were then grouped into subthemes, which created the facets of the four themes described in the Discussion section (detailed in online supplemental file 2). Due to the limited number of meetings that could be attended in the time frame, the data from meetings were used to triangulate the interview data rather than create their own themes. The findings have been presented back to the Trust’s leadership team.

10.1136/bmjoq-2022-002049.supp2 Supplementary data

**Table 2:** Traits of a Just Culture

	Positive traits (Just Culture)	Negative traits (Blame Culture)
Professional atmosphere	Open, trusting, supportive.	Fear, cautiousness, ‘club culture’.
Attitude to mistakes	Acceptance that mistakes will be made, systems in place to guard against human error.	Shame, embarrassment.
Attitude to reporting mistakes	Tolerance of human error, staff encouraged or rewarded to report mistakes.	Expectation of infallibility.
Focus of investigations	Organisational factors, improvement.	Individual culpability.
Support provided during investigation	Staff believe they will be supported by seniors. Staff expect to be viewed as a professional who behaved with no malicious intent.	Staff do not believe they will be supported by seniors. Staff expect to be viewed with suspicion and have their capability questioned.
Outcomes of investigation	Identify contributing factors. Organisational factors will be addressed and communicated. May be recommendations for further training.	Individual innocence or guilt. No consideration or communication of organisational factors.
Treatment of blame	Recognises that majority of human errors will be mistakes and blame is not appropriate. Recognises unacceptable behaviour such as deliberate action and gross negligence.	Blame culture—blame is prevalent and individual culpability will be suspected until evidence suggests otherwise. No-blame culture does not recognise and address unacceptable behaviour.

### Patient and public involvement

The only patient involvement in this study was the presence of two patient representatives at one of the Trust meetings observed.

### Research team and reflexivity

The two first authors, one male and one female, conducted the interviews and observed the meetings. Both are undergraduate MS with previous degrees (one with a BA and one with an MSc). One researcher has 5 years’ experience working in Transformation in a large private sector corporation, with a particular expertise in landing ways of working projects. They had no previous knowledge of Just Culture, however believed the NHS culture could be improved. The other served in the armed forces for 17 years in a division where Just Culture has been successfully implemented and is an advocate of Just Culture. Both have been trained in conducting research by WMS.

Prior to the interviews the participants did not know the interviewers apart from their occupation. The MS interviewed had not worked with the interviewers previously, but were known to each other on the course.

## Results

### Just Culture

Of the 20 people interviewed, only three had heard the term Just Culture before—both of the managers and one of the consultants. Of these three, one used Reason’s definition^5^ and two used variations of Dekker’s restorative approach.^15^ Notably, the two consultants who were employed as RCA Leads were unfamiliar with the term.

Despite the lack of familiarity with the term, all interviewees expressed a preference for a working environment with the positive traits of a Just Culture as detailed in table 2, indicating that staff would welcome a Just Culture. While interviewees stated that many of the positive traits were present in the Trust, there remained a lack of confidence among junior doctors in the clinical incident review process.

### Investigations: staff perspectives

The vast majority of the MS, F1-ST2/IMT2 (recently qualified, RQ) and ST3/IMT3+ (registrar, REG) were not familiar with the Trust’s guidelines regarding how clinical incidents are handled. The lack of understanding and perceived lack of transparency of the investigatory process by some interviewees were cited as causes of uncertainty regarding how staff would be treated.

I think a lot of junior doctors, in particular, don’t know the process. So, they have no idea what to expect and they’ve never been through anything like it, and it can be really, really, really scary. (REG 1)

Half of these interviewees showed concern regarding the negative impact of being investigated, regardless of outcome.

I remember hearing from one of the consultants that when you’re under investigation, even if it ends up and you’re innocent, the time that the, the stress you lived during the time of investigations causes a lot of problems. Even your family will notice that your mental life will be affected. (REG 2)

Concerns raised by junior doctors and MS related to whether they would be treated fairly if investigated included:

- Feelings that organisational factors will not be appropriately considered.
- Fear of being scapegoated with four interviewees citing the case of Dr Bawa-Garba.^20^
- Fear of racial discrimination.

Several junior doctors mentioned that familiarity with colleagues or the investigator might affect the investigation; some in terms of how their colleagues would perceive them during an investigation, and some in terms of whether the Trust would support them.

[Interviewer]: How would you feel if you were investigated on say your first week within a new department? If you didn’t have that relationship?

[RQ]: I would feel terrible to be honest because no one would have known me at that point of time. […] So if I don’t have anything, relationship during the first like, I mean, if I didn’t know them at all, then I would not feel secure and I would be thinking of different ways to have my back there. (RQ 1)

### Learning Culture

**Figure 1:**
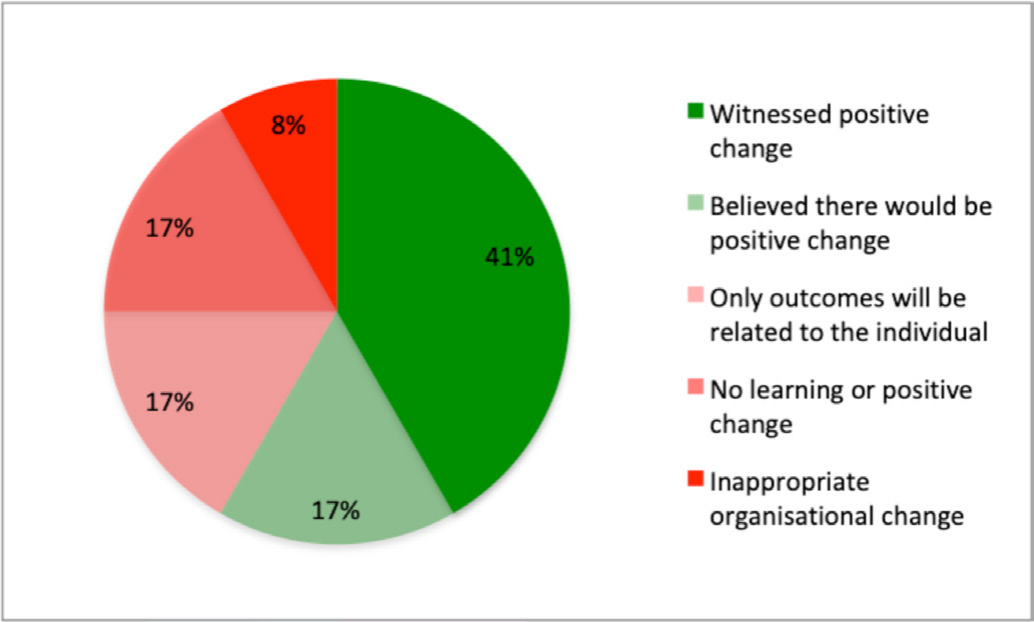
Breakdown of interviewee’s views on the outcomes of investigations.

The majority of interviewees believed that investigations produce positive changes to patient care (figure 1). However, one interviewee recounted how, in a different trust, inappropriate organisational change was made, convoluting working processes when individual training should have been implemented. It is worth noting we observed one TLIR meeting in which attendees focused on avoiding making inappropriate changes. Two interviewees stated they firmly believed no learning would come from investigations, believing the outcomes to be too vague and poorly communicated.

One of the critical points, so when a patient safety incident happens, you don’t know what, you don’t know what’s happening. How’s it been reported? What’s the final thing? How has it been fed into… clinical practice? Do all the nurses need to know about it or the doctors or physiotherapists? (CONS 1)

There was notable disparity between the views of consultants who were RCA Leads and other interviewees when the communication of outcomes was discussed. RCA Leads stated that a range of communication methods would be used including face-to-face feedback to individuals. The majority of the other interviewees, however, reported a heavy reliance on technology with meeting minutes, newsletters and reports being emailed to staff. Fewer interviewees reported face-to-face feedback via team meetings and handover meetings, but the majority of interviewees favoured these methods.

### Investigators

Interviews with managers and consultants revealed that not all investigators and RCA Leads were formally trained in conducting investigations. Those who had not received formal training highlighted informal training having participated in previous investigations prior to taking a lead role themselves. One consultant did state that a colleague within the Trust has identified a training course and is attempting to establish a core group of trained investigators, but that this initiative is at an early stage of development.

### Meetings

TLIR meetings displayed many traits of a Just Culture. Meeting participants did not use the names of staff involved in clinical incidents and discussions of incidents would usually begin with the chair highlighting that the aim was not to attribute blame, but to understand the situation and learn where patient care could be improved. The focus was placed on the ‘what’ and opposed to the ‘who’ and attendees attempted to identify organisational factors that may need addressing. M&Ms observed dissociated the situation from staff involved, which protected individuals from identification and blame, and organisational factors at a team and directorate level were discussed.

## Discussion

### Theme 1: Just Culture—what is it?

The attitudes of interviewees towards the traits promoted under a Just Culture are a key enabler that indicates that the promotion of this approach would be welcomed by staff. However, unfamiliarity of staff with the concept of a Just Culture is likely a current barrier to its adoption. Those familiar with the term provided three different definitions of Just Culture implying there may be no agreed definition within the Trust. The multiple definitions provided by NHS Improvement within its guidance may be a contributory factor. The plurality of definitions among seniors and lack of awareness among most of the staff suggest there has been limited discussion among staff regarding what a Just Culture means to the Trust.

### Theme 2: investigations—staff insecurities

The concerns raised by junior doctors and MS regarding clinical incident reviews and the investigatory process echo many of those raised in the Williams^8^ and Hamilton^9^ reviews. It was noted that junior doctors were under-represented at meetings observed, with the TLIRs observed having no doctors below consultant grade in attendance. The processes and behaviours that were observed, particularly at TLIRs, that aligned with the principles of a Just Culture suggest that the insecurities expressed may not reflect reality within the Trust. The unfamiliarity of the investigatory process may present a barrier to the adoption of a Just Culture and contribute to interviewees reporting concerns about insufficient consideration of organisational factors, discrimination and scapegoating. Improving the transparency of the process, particularly the conduct of TLIRs, has the potential to offer important reassurance to junior doctors.

Familiarity with colleagues was highlighted by REGs, RQs and MS as a protective factor that provided reassurance that they would be treated fairly during investigations. Thirty-three per cent of REGs and RQs interviewed highlighted the first few weeks in a new job as an unnerving period as they attempt to establish relationships and a professional track record. The frequent rotations during junior doctor training therefore create a barrier to establishing a Just Culture within a single trust as juniors regularly feel a heightened sense of insecurity at the start of new postings.

### Theme 3: Learning Culture

There were numerous instances observed when lessons were identified on an individual and organisational level during TLIR meetings, yet a sizeable minority of interviewees believed that investigations do not effectively drive improvement within the Trust. The proportion of staff interviewed who believed that investigations drive improvement (57%) is similar to the proportion of staff who were confident that their organisation would address their concern (59.6%), as reported by the NHS Staff Survey 2021. The fact that two in five members of staff doubt that reports and investigations will drive improvement is concerning.

The negative perceptions of either a lack of feedback from incident reports and reviews or feedback being received via impersonal and ineffective methods, such as newsletters and emails, are a potential barrier. Interviewees’ preference for more personal feedback via discussion echoes the findings of Sujan,^21^ who also found that use of discussions improved staff’s engagement with the safety organisation as they felt listened to and able to contribute to improving patient safety.

Both Reason and Dekker highlight that the desire to learn from incidents and improve is a key motivator for staff to engage with the mechanisms of an organisation’s safety culture.^5 15^ It is crucial that the Trust effectively communicates the outcomes of its investigations and incident reviews, both to aid the communication of lessons and implementation of changes, and also demonstrate to staff that reports and reviews drive improvement.

### Theme 4: investigators

The multiple definitions of a Just Culture, lack of awareness of a Just Culture and lack of formal training for investigators at the Trust are all barriers that have the potential to create variability in how staff involved in clinical incidents are treated. Furthermore, interviewees also reported an absence of formal human factors training among senior doctors. This may lend some credence to the perception by some REGs and RQs that organisational factors are not given sufficient weighting during investigations.

The effort of staff at the Trust to establish a formally trained incident investigation team is a promising initiative. Sussex Community NHS Foundation Trust^22^ reported a number of benefits associated with its full-time investigation team, including improved timeliness of investigations, implementation of outcomes and staff education.

### Limitations and further work

The number of interviews and meetings observed was limited by project duration and data collection for both interviews and ethnography stopped before the point of theoretical saturation.

The limitations of the cohort of interviewees means that results cannot easily be extrapolated to the wider workforce. The exclusion of other healthcare professionals, for example, nurses, from this study’s interviews was a significant limitation and may have led to positive bias in the reported perception of the culture.^23^ Differences in levels of exposure to clinical error and perceptions of how their regulatory bodies handle investigations may place varying influences on the willingness to report and perceived barriers for other healthcare professionals. While this study failed to capture the perspectives of nurses via interviews, ethnographic observation did capture the attitudes and behaviours of nurses and midwives to clinical incidents and mortality in both TLIRs and M&Ms.

While interviews attempted to capture a range of levels of experience among doctors, only one interviewee was from a surgical specialty. The use of mandatory triggers for reporting clinical incidents was a practice reported only by this interviewee and appeared to provide them with a higher degree of willingness to use official reporting mechanisms compared with interviewees from medical specialties. The lack of other surgical perspectives prevents us from determining whether this is a difference in practice between medical and surgical specialties in general, or specific to this interviewee’s directorate.

The observation of only five meetings is a significant limitation and provided only a glimpse at the review of clinical incidents within the Trust. This was, however, a valuable insight as it enabled the perspectives of interviewees to be analysed alongside the behaviours observed within meetings.

A mixed-methods study that includes a large survey would provide future research more confidence when analysing the extent to which a Just Culture exists within a Trust.

### Recommendations

#### Just Culture: agree your vision

The variation in attitudes and behaviours observed suggests that the Trust would benefit from continued discussion in order to precisely define what a Just Culture means to staff at the Trust.

#### Investigations: improve familiarity and transparency

The introduction of training into how clinical investigations are conducted, possibly during Trust induction sessions, would help improve familiarity among staff. The adoption and communication of a decision tree akin to the example in the NHS Just Culture Guide,^13^ or a charter, as suggested by the Being Fair Report,^14^ may also improve transparency. This would help inform staff where the ‘red lines’ are and the outcomes they should expect in different situations, which may help reduce feelings of insecurity regarding clinical investigations.

The inclusion of non-consultant grade doctors in TLIR meetings could help further improve familiarity with the process as well as adding insight from these doctors. That said, the presence of the clinician involved may produce an element of defensiveness, whereas the inclusion of a peer who was not involved may generate its own challenges. Further research would be valuable to provide evidence on this matter.

#### Learning Culture: a need for improved communication

Increased use of face-to-face meetings to communicate investigation outcomes may be useful to aid the implementation of organisational changes and also tackle negative perceptions among staff. Having a member of the investigation team communicate the outcomes at larger meetings could further enhance the effectiveness, as the presenter would be able to explain the rationale and answer any questions relating to the decisions made. This would also help build familiarity with and confidence in the investigatory process.

#### Investigators: incident investigation team

The adoption of a central incident investigation team has the potential to produce better quality investigations and more timely results for the Trust.^22^ This could further be enhanced by the Trust providing formal training in human factors, an initiative adopted by the General Medical Council.^24^ An incident investigation unit could help reduce feelings of insecurity held by staff regarding clinical incidents by providing:

- Incident management briefings during Trust inductions to improve understanding of Trust processes.
- Face-to-face feedback following investigations to aid implementation of outcomes.

## Summary

There is evidence of a fair incident management process within the Trust and staff interviewed expressed a preference for the traits of a Just Culture. Unfortunately, many remain insecure about being the subject of an investigation with potential barriers being: unfamiliarity with Just Culture; lack of transparency of investigatory processes; lack of training of investigators; and doubts regarding whether investigations lead to improvement. We believe the four recommendations made to improve culture, investigations and confidence among staff should be considered by all hospitals to improve their patient safety culture.

## Data Availability

No data are available. Due to ethical concerns and confidentiality reasons, supporting data obtained through semistructured research interviews with Trust staff and observations of Trust meetings cannot be made openly available. Semistructured interviews and observations of meetings were conducted under conditions of confidentiality and pseudonymity, thereby any raw data gathered through research interviews and meeting observations can neither be circulated nor made available to third parties. Contact email address for any data-related queries: s.brake@warwick.ac.uk.
