# Supplementary material for: How effectively has a Just Culture been adopted? A qualitative study to analyse the attitudes and behaviours of clinicians and managers to clinical incident management within an NHS Hospital Trust and identify enablers and barriers to achieving a Just Culture
Source: BMJ Open Qual. 2023 Jan 26;12(1):e002049. doi: 10.1136/bmjoq-2022-002049 (PMC9884909; doi:10.1136/bmjoq-2022-002049)
Supplement: Supplementary data [file bmjoq-2022-002049supp001.pdf]

Supplementary File: Interview Schedules

Medics Interview Schedule

Opening

A. (Introduction) My name is xxxxx and I am one of the researchers for this study. Thank you for agreeing to be interviewed. Just to let you know I may be looking at my notes to make sure I've covered all the points but I will still be listening and am very interested in what you are saying. Can I double-check your role/grade?

B. (Purpose) I would like to ask you some questions about your experience of working within the NHS. I'm particularly interested in your thoughts and feelings about the organisational structure, leadership, and the way that clinical incidents are managed.

C. (Motivation) We hope to use this information to understand the culture of the NHS and possibly identify good practice and also any barriers to improvement.

D. (Time Line) The interview should take about 30 minutes, is that OK?

E. (Recording) I will now be starting the recording and auto transcription, would that be ok?

F. (Consent) Have you had a chance to read the Participant Information Leaflet? Do you have any questions? Have you read the consent form? Do you consent to all aspects listed on the consent form?

Body

Part 1: Professional Atmosphere

1. Can you tell me about the atmosphere of your workplace?
[Open, trusting, supported vs Fear and cautiousness]

2. Can you tell me about the style of leadership you have experienced?
[Open, trusting, supported vs Club culture]

    a. How would you characterise the hierarchy?
    [Flattened hierarchy vs Vertical hierarchy, inability to question or challenge seniors]

3. How do you feel about the mistakes that you make at work?

    a. Why do you feel that way?
    [Acceptance that mistakes will be made vs Shame, embarrassment]

4. Do your working processes guard against errors that are made?
[Systems are in place that make it easy to do the right thing]

5. How do you feel about reporting mistakes that you make?

    a. What reporting systems are there in your trust?

b. How would you characterise the response of your seniors to your mistakes?
[Freedom to speak up without fear / tolerance of mistakes / encouraged or rewarded to report mistakes vs Expectation of infallibility]

Part 2: Conduct of Investigations

1. What would be the focus of an investigation into an error that resulted in death or harm to a patient?
[Organisational factors, improvement vs Individual culpability/focus, punitive]

2. Do you feel that you would be supported by your seniors and hospital management during an investigation?
[Seniors and colleagues support people involved in incidents]

3. How do you feel you would be viewed during an investigation?
[Professional with no malicious intent vs Suspicion, capability questioned]

4. What would you expect the outcomes of an investigation would be?

    a. Would the context and wider organisational factors be analysed?
    [Take a systems perspective / Identification of a range of contributing factors, recommendation for training vs Individual innocence or guilt]

5. How would results of the investigation be communicated?
[Organisational factors are addressed/communicated vs No consideration or communication of organisational factors]

6. Which sorts of errors should be investigated?
[All errors should be reviewed and possibly investigated vs only the most severe]

7. Should healthcare professionals be blamed for medical errors?

    a. What are your thoughts about considering an individual's intent?

    b. What are your thoughts about considering an individual's capability?

    c. What are your thoughts regarding whether an individual adhered to or deviated from Trust procedures?

    d. What are your thoughts regarding an individual who deviated from Trust procedures but thought they were doing so for good reason?
    [Don't accept unacceptable behaviour / Deliberate action and gross negligence vs Blame culture (blame for errors and mistakes) or No-blame culture (staff not disciplined for unacceptable behaviour – deliberate harm, recklessness, gross negligence]

Part 3: Just Culture Knowledge

1. What is meant by the term Just Culture?

2. What training have you received regarding a Just Culture?

3. Have you received training in human factors?

4. Have you read the NHS Improvement guide?

    a. What are your thoughts on the guidance?

5. Does your trust have guidelines on how mistakes will be handled?

6. Does your trust have a Just Culture policy?

Closing

A. (Close) I appreciate the time you took for this interview. Is there anything else you would like to discuss in relation to any of the issues that we have discussed?

B. (Action to be taken) A transcript of this interview will be made. You will be given a pseudonym in the transcript and all of your personal details will be removed. We will analyse your response alongside all other participants and alongside all other [Consultants/Registrars/Recently Qualified Doctors/Medical Students]. The report that we produce may include a quote from this interview, which would be attributed to a [Consultant/Registrar/Recently Qualified Doctor/Medical Student]. The report will not name the Trust.

Managers Interview Schedule

Opening

A. (Introductions) My name is xxxxx and I am one of the researchers for this study. Thank you for agreeing to be interviewed. Just to let you know I may be looking at my notes to make sure I've covered all the points but I will still be listening and am very interested in what you are saying. Can I double-check your role/grade?

B. (Purpose) I would like to ask you some questions about your experience of working within the NHS and as a leader. I'm particularly interested in the organisational structure, leadership, and the way that clinical incidents are managed.

C. (Motivation) We hope to use this information to understand the culture of the NHS and possibly identify good practice and also any barriers to improvement.

D. (Time Line) The interview should take about 45 minutes, is that OK?

E. (Recording) I will now be starting the recording and auto transcription, would that be ok?

F. (Consent) Have you had a chance to read the Participant Information Leaflet? Do you have any questions? Have you read the consent form? Do you consent to all aspects listed on the consent form?

Body

Part 1: Professional Atmosphere

1. Can you tell me about the atmosphere of your workplace?

   a. How did you envision the atmosphere to be?

   b. What influenced you when creating this vision?

   c. What steps did you take to encourage this atmosphere?
   [<u>Open, trusting, supportive</u> vs Fear and cautiousness]

2. Can you describe your style of leadership?
[<u>Open, trusting, supportive</u> vs Club culture]

   a. How would you characterise the hierarchy?

   b. How approachable do you think your senior clinicians and colleagues are?

   c. Do you feel you are an approachable senior to your juniors?
   [<u>Flattened hierarchy</u> vs Vertical hierarchy]

3. How do you feel about the clinical mistakes that are made at work?

   a. Why do you feel that way?

   b. Do you think that your clinicians feel the same way?
   [<u>Acceptance that mistakes will be made</u> vs Shame, embarrassment]

4. Does the trust have working processes guard against errors that are made?
[Systems are in place that make it easy to do the right thing]

5. How do you feel about reporting mistakes that you make?
[Freedom to speak up without fear / tolerance of mistakes / encouraged or rewarded to report mistakes vs Expectation of infallibility]

6. How do you want clinicians to feel about reporting mistakes?
[Freedom to speak up without fear / tolerance of mistakes / encouraged or rewarded to report mistakes vs Expectation of infallibility]

7. Do you think clinicians have this response?
[Freedom to speak up without fear / tolerance of mistakes / encouraged or rewarded to report mistakes vs Expectation of infallibility]

8. What have you done to promote that?

    a. What reporting systems are there in your trust?
    [Freedom to speak up without fear / tolerance of mistakes / encouraged or rewarded to report mistakes vs Expectation of infallibility]

Part 2: Conduct of Investigations

1. Who would typically conduct this investigation?

2. What training do they receive for this role?

3. What would be the focus of an investigation into an error that resulted in death or harm to a patient?

    a. Why would you want this to be the focus?

    b. What has influenced your view?

    c. What have you done to promote this?
    [Organisational factors, improvement vs Individual culpability/focus, punitive]

4. Do you feel that your clinicians would feel supported by your seniors and hospital management during an investigation?
[Seniors and colleagues support people involved in incidents]

5. How would you support staff?

    a. How would you support staff if they had been suspended?
    [Seniors and colleagues support people involved in incidents]

6. How do you think a clinician would feel during an investigation? What would you do to promote that?
[Professional with no malicious intent vs Suspicion, capability questioned]

7. How frequently would you consider an action like suspensions or limitations of duties,

    a. How would that impact the colleague?

8. What would you expect the outcomes of an investigation would be?

    a. Would the context and wider organisational factors be analysed?
    [Take a systems perspective / Identification of a range of contributing factors, recommendation for training vs Individual innocence or guilt]

9. How would results of the investigation be communicated?
[Organisational factors are addressed/communicated vs No consideration or communication of organisational factors]

10. Which sorts of errors should be investigated?
[All errors should be reviewed and possibly investigated vs most severe]

11. Should healthcare professionals be blamed for medical errors?

    a. What are your thoughts about considering an individual’s intent?

    b. What are your thoughts about considering an individual’s capability?

    c. What are your thoughts regarding whether an individual adhered to or deviated from Trust procedures?

    d. What are your thoughts regarding an individual who deviated from Trust procedures but thought they were doing so for good reason?
    [Don’t accept unacceptable behaviour / Deliberate action and gross negligence vs Blame culture (blame for errors and mistakes) or No-blame culture (staff not disciplined for unacceptable behaviour – deliberate harm, recklessness, gross negligence]

Part 3: Just Culture Knowledge

1. What is meant by the term Just Culture?

2. What training have you received regarding a Just Culture?

3. What training does the Trust provide?

4. Have you received training in human factors?

5. Have you read the NHS Improvement guide?

    a. What are your thoughts on the guidance?

6. Do you think clinicians are aware how mistakes are handled in the trust?

    a. How is this communicated to your clinicians?

7. Does your trust have a Just Culture policy?

    a. What guidance is it based on?

    b. What have you done to promote it?

Closing

A. (Close) I appreciate the time you took for this interview. Is there anything else you would like to discuss in relation to any of the issues that we have discussed?

B. (Action to be taken) A transcript of this interview will be made. You will be given a pseudonym in the transcript and all of your personal details will be removed. We will analyse your response alongside all other participants and alongside all other managers. The report that we produce may include a quote from this interview, which would be attributed to a manager. The report will not name the Trust.
