# Supplementary material for: How effectively has a Just Culture been adopted? A qualitative study to analyse the attitudes and behaviours of clinicians and managers to clinical incident management within an NHS Hospital Trust and identify enablers and barriers to achieving a Just Culture
Source: BMJ Open Qual. 2023 Jan 26;12(1):e002049. doi: 10.1136/bmjoq-2022-002049 (PMC9884909; doi:10.1136/bmjoq-2022-002049)

Supplementary File 2: Thematic analysis

Theme 1: Just Culture: What is it?

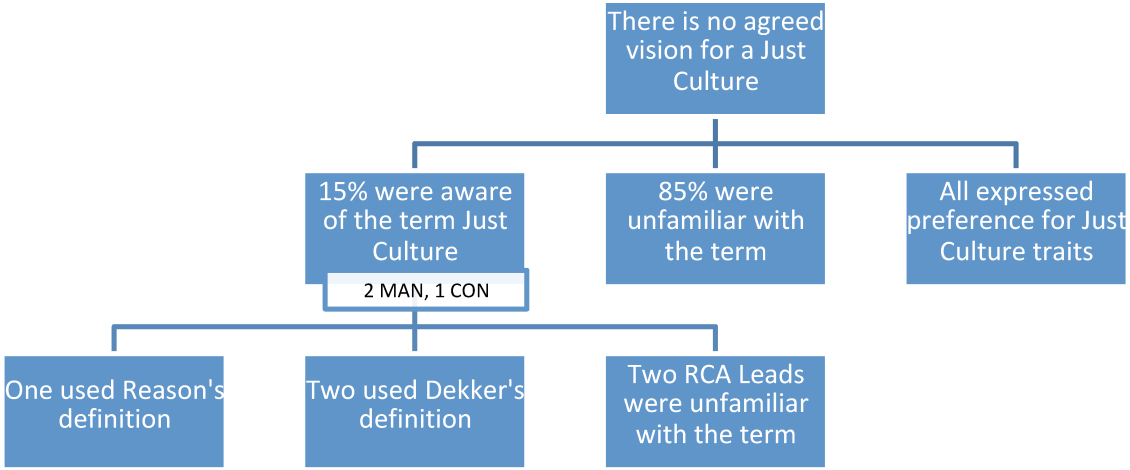

Theme 2: Investigations: Staff insecurities

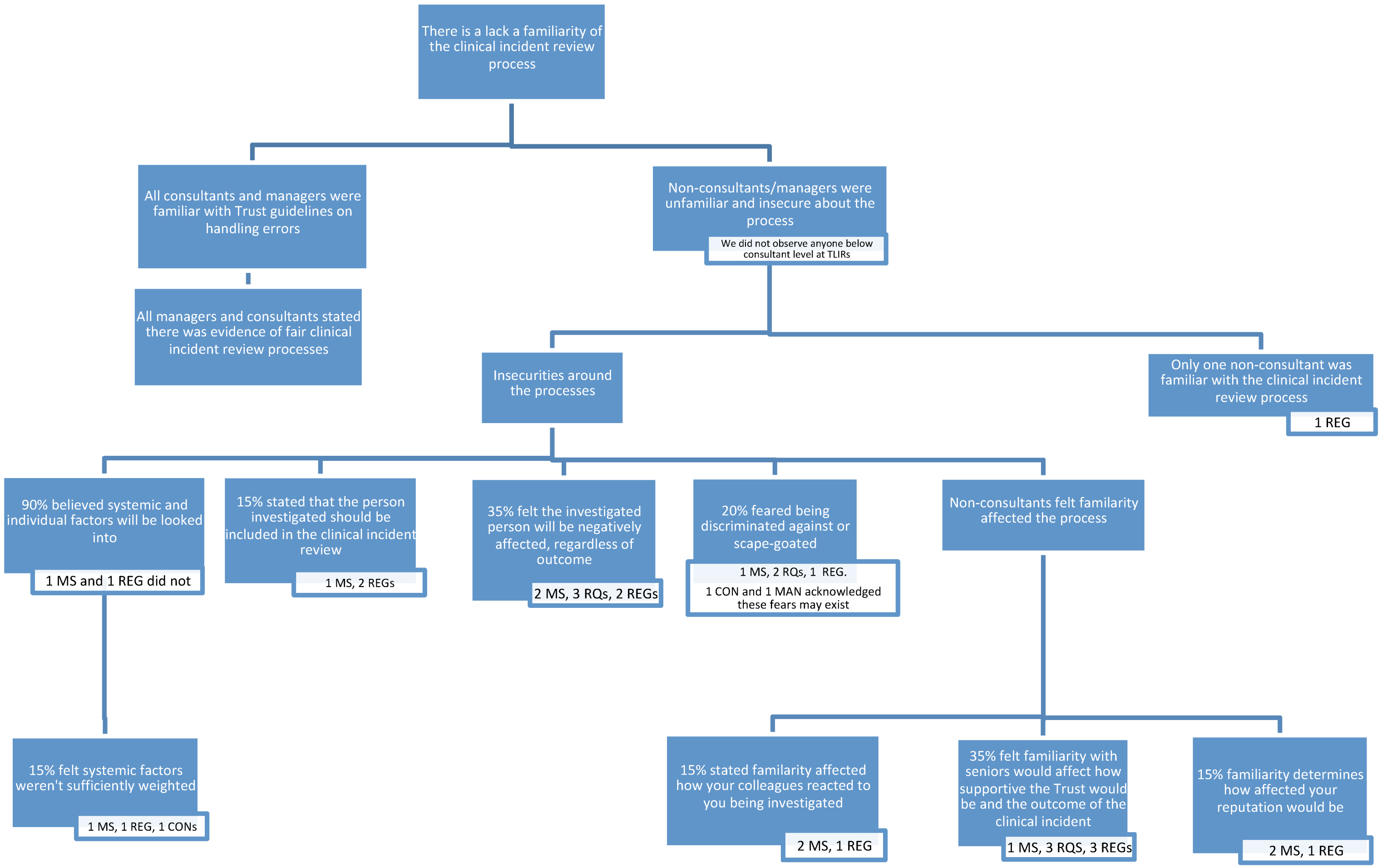

Theme 3: Learning Culture

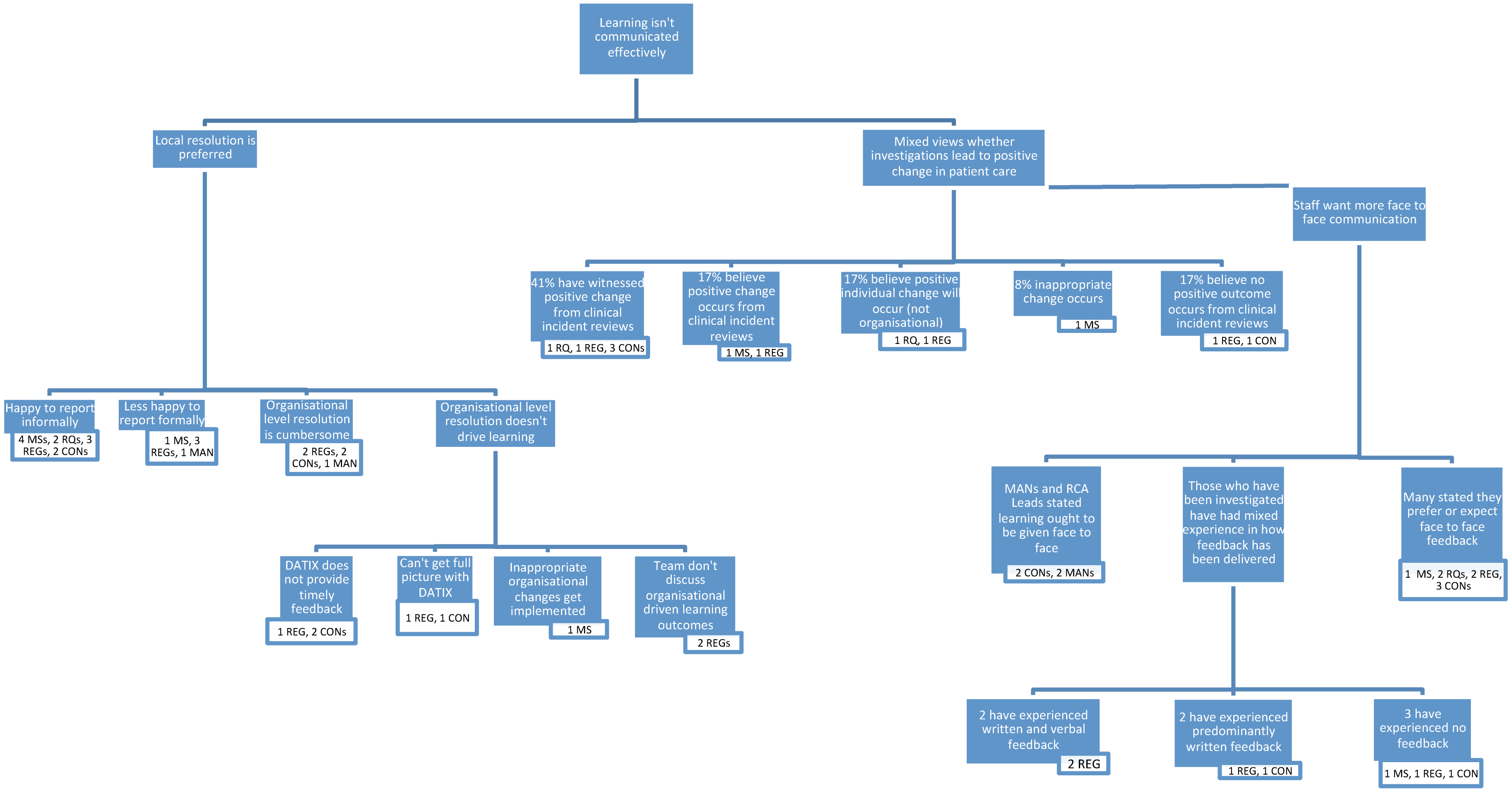

Supplement: Supplementary data [file bmjoq-2022-002049supp002.pdf]
